# The potential for multi-disciplinary primary health care services to take action on the social determinants of health: actions and constraints

**DOI:** 10.1186/1471-2458-13-460

**Published:** 2013-05-10

**Authors:** Frances E Baum, David G Legge, Toby Freeman, Angela Lawless, Ronald Labonté, Gwyneth M Jolley

**Affiliations:** 1 Southgate Institute for Health, Society, and Equity, Flinders University, GPO Box 2100, Adelaide, SA 5001, Australia; 2 School of Public Health & Human Bioscience, La Trobe University, 215 Franklin Street, Melbourne, Victoria 3000, Australia; 3 South Australian Community Health Research Unit, Flinders University, GPO Box 2100, Adelaide, SA 5001, Australia; 4 University of Ottawa, Institute of Population Health, 1 Stewart Street, Ottawa, Ontario K1N 6N5, Canada

**Keywords:** Primary health care, Health promotion, Social determinants of health, Health equity, Community health, Aboriginal health

## Abstract

**Background:**

The Commission on the Social Determinants of Health and the World Health Organization have called for action to address the social determinants of health. This paper considers the extent to which primary health care services in Australia are able to respond to this call. We report on interview data from an empirical study of primary health care centres in Adelaide and Alice Springs, Australia.

**Methods:**

Sixty-eight interviews were held with staff and managers at six case study primary health care services, regional health executives, and departmental funders to explore how their work responded to the social determinants of health and the dilemmas in doing so. The six case study sites included an Aboriginal Community Controlled Organisation, a sexual health non-government organisation, and four services funded and managed by the South Australian government.

**Results:**

While respondents varied in the extent to which they exhibited an understanding of social determinants most were reflexive about the constraints on their ability to take action. Services’ responses to social determinants included delivering services in a way that takes account of the limitations individuals face from their life circumstances, and physical spaces in the primary health care services being designed to do more than simply deliver services to individuals. The services also undertake advocacy for policies that create healthier communities but note barriers to them doing this work. Our findings suggest that primary health care workers are required to transverse “dilemmatic space” in their work.

**Conclusions:**

The absence of systematic supportive policy, frameworks and structure means that it is hard for PHC services to act on the Commission on the Social Determinants of Health’s recommendations. Our study does, however, provide evidence of the potential for PHC services to be more responsive to social determinants given more support and by building alliances with communities and social movements. Further research on the value of community control of PHC services and the types of policy, resource and managerial environments that support action on social determinants is warranted by this study’s findings.

## Background

In 2008 the Commission on the Social Determinants of Health (CSDH) recommended the need for health systems to be based on primary health care (PHC) and to be able to take action on the social determinants of health (SDH). These recommendations were reinforced by the World Health Organization’s Rio Political Declaration on Social Determinants of Health [1] and a United Nations Declaration on the Prevention and Control of Non-communicable Diseases [2]. Each saw comprehensive PHC as having a role in reducing health inequities through action on SDH. These recommendations built on earlier WHO statements on PHC and the Ottawa Charter for Health Promotion [3] and the subsequent series of global health promotion conferences (see [4]) each of which have stressed the need for a reoriented health sector and healthy public policy through intersectoral action. This paper examines the extent to which six case study PHC services in Australia took action on the SDH and the constraints they faced in doing so. The paper starts with an exploration of debates about the roles, function and ideological underpinnings of PHC in the past thirty years particularly in relation to tackling SDH.

The concept of PHC evolved during the 1970s, influenced by and influencing the basic needs approach to social development [5]. Informed by both the disappointments experienced in implementing the basic health services approach [6] and by the significant progress in improving health in China in the 1960s and 1970s, as well as by the achievements of many small, mostly NGO-inspired, community-based healthcare initiatives [7] including the Aboriginal community-controlled movement in Australia [8], the World Health Organization (WHO) and the United Nations Children’s Fund (UNICEF) elaborated the strategy of PHC as the means to achieve ‘Health for All’ by the year 2000. The WHO Alma Ata Declaration [9] saw PHC as a focus for community action to tackle the underlying determinants of health thus situating PHC within a broader social movement designed to reduce inequities and improve living conditions for whole populations [6,10-12].

This social view of health was not new in the 1970s and has roots going back to the nineteenth century. In Europe both Engels [13] and Virchow [14] recognised that disease affected the poor more than the rich and that social conditions were vital in this relationship. A similar recognition was evident in Latin America where the social medicine movement, also with its roots in the nineteenth century, placed emphasis on the social basis of health [15]. The Marxist argument that health inequities have their origins in social class difference and the reproduction of these classes through capitalism has been applied to contemporary society [16]. This body of thinking argues that understanding the SDH requires an analysis of how class-driven poverty, and the alienation and relative powerlessness that goes with it, sets the scene for constellations of vulnerabilities and behaviours which contribute to multiple diseases. The role of the health sector in addressing these conditions of vulnerability, and the behaviours and diseases to which they give rise, requires a comprehensive approach which goes beyond service provision to address social and economic inequities.

Despite the strong case for a comprehensive PHC approach it was challenged very soon after the Alma Ata Declaration was published. Walsh and Warren [17] argued that “until primary health care can be made available to all, services targeted to the few most important diseases may be the most effective means of improving the health of the greatest number of people” (p. 973). This technical argument ignores the sociological arguments for comprehensive PHC. The debate between selective and comprehensive PHC reflects deeply rooted perspectives on how health is created and why it is distributed unevenly in societies. The logic of a selective PHC is rooted in disease and behavioural epidemiological understandings which do not attempt to account for the social and political basis of health and which align more closely with a biomedical than social approach to health. Biomedicine sees diseases as residing in the bodies of individuals and so actions to address them are directed at curing the individual or persuading them to reduce their risk factors for disease [18], emphasizing curative and rehabilitative therapies. The biomedical paradigm has established a pervasive and powerful position [19,20] and it continues to dominate in health systems around the world. This position of privilege is reinforced by the individualism that is at the heart of neo-liberalism [10]; and, indeed, the rise of biomedicine in the early 20^th^ century has been argued as due partly to its ideological alignment with industrial capitalism [16]. The hegemony of these ideas was argued powerfully by Tesh [21] when she showed that the “hidden arguments” in many public health debates are actually ones of ideology in which the dominant ideology of individualism remains hidden and is presented as taken-for-granted knowledge.

Applying Tesh’s [21] insight to the debates about PHC suggest that the seemingly “apolitical” perspective of selective PHC and its focus on treating a few diseases with largely medical therapies also serves to maintain the status quo in terms of resource and power distribution. Health politics lie in controlling the costs of publicly funded health care, managing the conflicts between health professionals for control of health care and developing efficient management models [22]. In this context the potentially redistributive role of PHC in dealing with underlying causes is inevitably controversial and contested, uncomfortably positioning PHC services as one of the key meeting points between biomedicine and sociological understandings. Comprehensive PHC may be opposed because it is perceived to be (and may actually be in practice) threatening to the social, economic and political status quo given that its aim is to reduce health inequities by challenging the inequitable structures which underpin them [16,23]. This position raises many questions for the ways in which PHC services are able to respond to SDH and what factors support or impede responses. The mixed progress in improving health under the western bio-medical model has led to continued debate about styles of PHC. Thus increasing burdens of chronic disease (which are complex to manage) in countries at all levels of development [24,25] and the increased inequities in wealth and health over the last two decades [26-28] indicate that comprehensive PHC is needed as much today as it was in 1978.

Despite this support for comprehensive PHC, commentators have argued that PHC (including in Australia) has become more selective as an individual and disease based approach has overtaken the original 1978 vision [29-31] and that behavioural paradigms dominate despite acknowledgement of the importance of SDH [32]. In Australia successive Federal governments have chosen to give resources and policy attention to developing primary medical care rather than strengthening comprehensive PHC. There has also been a progressive withdrawal from the community managed services introduced in the 1970s by the reformist Whitlam Labor government to PHC services that are directly managed by the state health departments, with the exception of the Aboriginal community controlled sector. The adoption of selective rather than comprehensive PHC models is also underpinned by the lack of any serious research or policy attention to what comprehensive models of PHC could or should look like in practice. In the 2008 Now More Than Ever WHO report on PHC, there is no attempt to articulate or describe possible practices of comprehensive PHC. Nor does the report suggest how such practices could evolve from more selective PHC models. This presents PHC practitioners and managers with dilemmas [10,33] reminiscent of Hoggett, Mayo and Miller’s [34] analysis of development social work in the UK where they noted the uncomfortable position of workers between the neo-liberal policies of the state and local communities. Comprehensive PHC implies challenge to the status quo of unhealthy structures, which means that PHC workers implementing this strategy at a local level will come against the power structures that maintain and reproduce the status quo. Practicing PHC in a way that does challenge power structures has rarely been considered in the literature. PHC research has focussed on “slices” of work conducted in PHC centres rather than the responses of workers to these contradictions [35,36]. The only insights in the literature come from books considering these tensions in PHC. Broom [37] explores the contradictions between feminist ideology and the operation of state funded women’s health centres and demonstrates the very significant dilemmas that were posed for services trying to reconcile the contradictions in their practices. Legge et al’s [38] analysis of 185 published accounts of PHC practice stressed the importance of community involvement, collaborative local networking, vertical networking, integrating a concern for the local (“micro”) level and longer term, macro issues and the importance of change consciousness. Baum, Fry and Lennie [39] described the Australian community health movement and Baum [40] details the experience of PHC in South Australia. Both highlighted very similar elements for best practice to those identified by Legge et al. [38]. A strong community health sector working on principles of comprehensive PHC operates in some provinces in Canada (see http://www.cachc.ca/) and Lefkowitz [41] describes the community health movement in the United States highlighting its roots in the civil rights and social justice movements. This small evidence base does suggest that practicing comprehensive PHC requires PHC workers to be reflexive about the role of a PHC service when it practices in a manner that extends beyond delivering services to individuals and seeks to empower people and communities to change the conditions that give rise to poor health in the first place. Giddens [42,43] stresses that a fundamental feature of modernity is the reflexivity of social life and that there has been an acceleration in the processes of social reflexivity. These processes extend to professional practices such as those associated with comprehensive PHC, which requires engagement with individuals and the structures that shape their chances for health. Thus it is important to determine PHC workers’ awareness of how social conditions constrain and promote health and how they perceive these conditions affecting their practice and how their practice is itself constrained by structures.

Our research builds on the small literature on comprehensive PHC and examines the dilemmas of comprehensive practice and PHC workers’ reactions to these dilemmas. It reports on research conducted in six multidisciplinary Australian PHC services in relation to the following questions:

1. To what extent do the activities of PHC services encourage systematic responses to SDH?

2. What barriers exist to systematic responses to SDH?

3. What implications do the findings have for understanding the potential for effective implementation of comprehensive PHC?

## Methods

### Study history and context

In Australia the responsibility for PHC is shared between the Federal government and the governments of the six states and two territories, resulting in PHC operating in two parallel systems. Primary medical care largely operates as a fee-for-service scheme for which patient fees are covered by the Federal universal public health insurance scheme Medicare. Each State and territory government also provides PHC services which were originally established as part of a Federal Community Health Program [44] in 1972 but since 1975 (when the Federal scheme ceased) have developed differently in each jurisdiction [45,46]. PHC services have also been provided by Aboriginal controlled health services from the 1970s [8,32,47].

### Study sites

Data were collected from six sites in the first year of a five year research partnership which each service agreed to participate in. The study sites are an Aboriginal Community Controlled Organisation (Central Australian Aboriginal Congress), a sexual health NGO (Shine SA), both of which have requested to be identified in publications, and four services funded and managed by the South Australian government, which have been anonymised. An overview of the characteristics of the services is provided in Table 1. All the services had client eligibility criteria (being Aboriginal in the case of Congress and Service D) which meant that clients were overwhelmingly from disadvantaged backgrounds. The services were selected to provide a range of examples of Australian PHC services and because the research team had an existing working relationship with the services that made a detailed five year study feasible. Ethics approval was received from the Flinders University Social and Behavioural Research Ethics Committee and the Aboriginal Health Research Ethics Committee (South Australia).

**Table 1:** Characteristics of the six case study PHC services, 2010

	**Approximate # of staff (FTE)**	**Budget (p.a.)**	**Main source of funding**	**Governance**	**Examples of disciplines employed**
Service A	16 (13.5)	$1.2m	SA Health	State funded and managed	Social worker, nurse, speech pathologist, occupational therapist, dietitian, cultural worker, lifestyle advisor
Service B	26 (20)	$1.1m	SA Health	State funded and managed	Medical officer, lifestyle advisor, PHC worker, podiatrist, nurse, speech pathologist
Service C	36 (22)	$1.7m	SA Health	State funded and managed	Nurse, dietitian, speech pathologist, psychologist, occupational therapist, cultural worker, social worker
Service D	12 (10.8)	$0.5m	SA Health	State funded and managed	Aboriginal health worker, PHC worker
Congress	320 (188)	$20m	Dept. of Health & Ageing	Aboriginal community controlled Board	Medical officer, psychologist, social worker, youth worker, midwife, nurse, Aboriginal health worker, pharmacist
Shine SA	100 (55)	$6.1m	SA Health + Dept. of Health & Ageing	Non-government organisation with governing Council	Medical officer, nurse, counsellor, workforce educator, community health worker, disability worker, Aboriginal educator, multicultural worker

### Interviews

Key informant semi-structured interviews [48] were chosen as a method to gather expert perspectives on action and constraints on action on SDH in different PHC services because they allowed for nuanced discussion about the extent to which services were able to take action on the SDH. At each site, 7–15 in-depth semi-structured interviews were conducted with managers, practitioners and administrative staff. Data were collected on how respondents understood the role of PHC services in responding to SDH and the ways in which these understandings shaped practice. Interview schedules were developed by the research team and piloted on three practitioners and one manager. Example questions included “What does the service do to support action on the social determinants of health?”, “What examples of this work can you describe?”, and “Who do you work with to tackle the social determinants of health?”

Interviewees were purposefully selected to maximise diversity in disciplines and to ensure all key viewpoints were included. Specific disciplines were requested from each site such that staff interviewed reflected the overall spread of disciplines employed at the six sites. In addition, six regional health executive staff and two representatives from the central office of the state health department were interviewed. Interviewees were recruited through direct invitation from the research team for managers, regional health executives, and state health department staff, and invitation via managers for staff at the services. A total of 68 interviews were conducted by the research team. The team members were academic researchers who held experience in working in and/or conducting research with primary health care services, but were not current clinicians. Interviewees were provided with an information sheet in advance and were asked to complete a consent form prior to the interview commencing. No one who the research team approached declined to participate. Interviews were transcribed in full by an external service then checked by a member of the research team. Transcripts were de-identified, and the anonymity and confidentiality of individual respondents was ensured. Data collection was ended once the selected 68 interviews were completed and the research team agreed data saturation had been reached.

### Data analysis

Thematic analysis was conducted by the authors. Codes were developed, discussed and revised during regular team meetings ensuring rigour through constant monitoring of analysis and interpretation [49]. Preliminary analysis revealed both emerging common themes covering underlying principles, activities, operating environment, and desired outcomes as well as some divergent views, constituting a ‘meaningful range’ [50]. The first author then finalised the codes concerning SDH, and lead the development of the categories and analysis presented here. Patterns and relationships between the data were identified and examined. Analysis then progressed from description, to explanation or interpretation of the patterns and their broader meanings and implications [51]. Emerging findings were presented to staff meetings at the PHC services and presented and discussed at project meetings attended by all investigators and PHC service stakeholders to check the validity of findings and to seek any alternative explanations. Quotes to present were selected when they provided an example of a common viewpoint or theme, or if they provided unique information from a particular service or viewpoint. The final drafts of all papers emanating from the study are sent to the managers of the PHC services participating in this study in order to seek their comment and agreement to submission of the paper. The data collection and reporting adheres to the RATS guidelines on qualitative research (http://www.biomedcentral.com/authors/rats).

## Results

### Conceptualising, understanding and developing practices in relation to social determinants

Our discussions with the PHC workers suggest that the term “social determinants” incorporates a heterogeneous group of factors that differ in terms of scale (global, national, regional, local), the extent to which they are contested and the extent to which PHC services can respond directly to address them. Acting on these complexities requires reflection from the workers and managers in regard to how their own work practices are able to take account of and, in some cases, act to mitigate the impact of these determinants. Practice was shaped significantly by the context of the organisation and the broader health system. These organisational environments, in the main, operated to restrict the agency of workers with respect to SDH; below we examine their accounts within this context.

#### Understanding

Respondents discussed how they understood SDH and the impact they perceived them having on the populations they worked with. Most responses indicated that they recognised the importance of SDH as shown by this comment:

… *and the structures that support health and wellbeing. Whether that is safe roads, safe environments, no disadvantage, no great pockets of poverty so that people do have access to a good quality education system and all the stuff that is supportive of good health*. (Regional Health Executive)

Often the workers linked statements about SDH to their detailed understanding of the community in which they worked as this nurse noted:

*Well what we know of course in this specific area is we’re one of the lowest socio economic areas in Australia, let alone in South Australia, and so we are aware transport, lack of cars, funding is a huge thing. We’re on the fourth generation of unemployment.* (Nurse, Service A)

Most statements about SDH were cast in light of the heavy burden of disadvantage experienced in the communities served. This was most acute in the Aboriginal services.

*Our clients have multiple issues. It could be financial, it could be housing, education, and all that can contribute to bad health in ways where if you don’t have a proper income, you're not getting proper food. I mean just not being educated, you're not going to know how to read signs, maybe not go into services because you can't even talk to the receptionist.* (Worker, Service D)

Most staff at all the services were aware that problems with SDH translates into illnesses and so the populations they serve have a high burden of illness;

*We’ve just got the highest social determinants of ill health … And I guess our contribution is seeing people that don’t necessarily access other services. Each client we see has got multiple illnesses, from younger people to old people, who just really show a burden of ill health from early age to later age.* (Medical Officer, Service B)

Workers showed a clear understanding of the limits that adverse social conditions placed on the ability of the people they worked with:

*People will say “I’m skipping meals because I’ve run out of money” or “I’m eating the cheap sausages because they’re cheap rather than having a piece of steak”, stuff like that. So a real connection to their money and the kind of food you eat. Also lots of clients are female and they have young children and so their children come first. So they’ll say “Yeah I buy all this fruit at the beginning of the week and my children eat it, which I want them to do and so I won’t eat the fruit”* (Lifestyle Advisor, Service A)

Through these comments workers were clearly indicating the limited agency their clients were able to exert in the face of the structural constraints of unemployment and poverty, including the last comment from a worker whose main remit was to promote healthy behaviours. They provide very explicit pictures of the ways in which a life lived in poverty makes it hard to make healthy choices. The material circumstances of life were also linked to mental health as in this example:

*Housing is a major issue. Often you can’t start unlocking the client’s depression if the home environment is not - and the social determinants of health is a really big issue in that respect, it’s just massive.* (Manager, Congress)

A minority of staff, typically younger and less experienced showed less awareness of the work of their service in responding to SDH. For example:

*There is work that’s being done here, not particularly with me but I don’t quite know the extent of that. But I know the counsellors and the social workers have roles to find people housing and help people with those sorts of things; writing letters for people, but I don’t know the extent of those things.* (Worker, Service A)

Most of the staff showed a good understanding of the ways in which SDH affected the communities they worked with and saw that they made their work more complicated and challenging. The interviews suggested the staff were generally reflexive about the social and economic constraints on people’s lives and the impact that these constraints have on their health and also on the ability of PHC services to fully respond to the issues people have to deal with.

#### Developing practices to respond to SDH

Respondents commonly emphasised using a holistic approach in which the circumstances of people’s lives were central to how they were treated by the services.

*We don’t just look at the medical model, we look at the whole person. Not just their medical problems, but social, economical, environmental, all those things that affect people, like education, income. So we don’t just focus on one little thing, we try and just see the whole person and the whole picture, not just a small part of it.* (Aboriginal Health Worker, Congress)

A similar picture was presented by a member of SHine SA:

*I think if you went and talked to someone in our clinic out at [suburb], then they would be saying holistically there are a whole lot more problems, issues, when they see a client. Other things impact a whole lot more on their sexual health and their general health, like their drug and alcohol use, or their homelessness, or being in prison when we run the clinic at the prison* (Nurse, SHine SA)

Some also noted that adverse social determinants can make intervention very difficult:

*You can’t do therapeutic counselling with someone who has no home or has got the bills piled up and can’t pay the bills.* (Counsellor, Service C)

For others it was about constantly keeping in mind the limitations people faced in their lives and, where possible, linking them in to services that may help them:

*So while it might not be that we’re actively involved necessarily in being able to influence those social determinants of health directly ourselves, often it is about linking people in with services who could assist them with that and then working with those services.* (Psychologist, Service C)

All of the services offered group activities. These included groups for people with a particular illness such as diabetes or depression, those defined by a social issue such as domestic violence, or with risk factors such as exercise or eating behaviours. These groups were generally designed with an appreciation of the limits people have on their lives and in some cases challenged social stereotypes as this nurse noted:

*The Dads’ group looks at social change about the role of the father. It encourages fathers to fight against the stereotypes of what is a father, and to encourage [them] to be more involved.* (Nurse, Service C)

Another example is the statewide program *Community Foodies*[52] which involves local residents in a peer education and support program to promote healthy eating based on a longstanding concern with access to low cost, healthy food.

#### More than a place to deliver services

The services were more than a place in which health professionals see individuals. They were also conceived of as community spaces and, to a varying degree among the services, places which were also the site of community action. This openness to community also signifies an understanding of the importance of community connectedness as itself a SDH. The importance of space has been noted in a previous study of community health centres [53] which noted how space can “permeate into social relationships, experiences of health and identity and connections to a sense of community” (p. 1874). This appears to remain true in our study sites. This was shown most forcefully in the case of a men’s service within Congress which was seen as a safe and welcoming space and one which could provide social support:

*So you can come in and have a yarn with your mates and have a shower and wash your clothes, just chill out for a while. A lot of men actually like to come in here because they said there’s no humbug here. So if they’re living in an area where there’s lots of drinking, they can’t sleep and that sort of stuff so it’s just a hassle free place. We don’t get in anyone’s face and they just come and take it easy and do what you have to do.* (Manager, Congress)

This manager highlights the importance of making the men feel comfortable as a first step to them using PHC services. SHine SA were also aware of the importance of creating a welcoming space which could be used for community activities:

*The office at [suburb], for example, has a resource library and coffee and internet café. So the view is, it’s a drop in centre. And also if consumer groups, community groups want to use the premises for other meetings and things like that, all of those offices are available to that.* (Board member, SHine SA)

Service C reached out to people with mental illness who lived in local boarding houses by creating a community garden, which encouraged these clients to feel comfortable with the service and see the staff as approachable as well as a place where people could form friendships and networks:

*It is such a great way to get people involved in something and essentially they are contributing to something that they see changes over time - growing plants and vegetables. But they are also socialising, they are also interacting with health professionals, like myself.* (Worker, Service C)

Service D used their centre to hold lunches, which once again enabled people to make social connections and become familiar with services, as one of their workers explained:

*…. It’s an opportunity for us to promote our health professionals in this area as well so that the community can put faces to names…. So Housing comes down here so they can put a face to their names or their workers there, all the other non-government organisations come down* (Aboriginal Health Worker, Service D)

These examples indicate how PHC service staff see their role as going beyond the provision of services to one in which they create welcoming spaces in which people feel comfortable and provide opportunities for social connection, itself an important SDH and link them to other (non-health) services and programs. In this way they were increasing opportunities for clients to use services and especially clients who faced barriers to using services for cultural, gendered or other social reasons.

#### Advocating for access to services

Staff described how they were able to act as advocates for individuals and assist them in gaining access to housing, social security benefits, legal advice, or helping women to leave violent situation. For Service D the support role reflected the history of violent situations distrust between Aboriginal people and state organisations and was expressed by a worker who saw their role as “advocating and supporting clients and building relationships to break down barriers such as distrust of government services.” A worker at Congress noted the importance of advocacy to other services for their clients when they said:

*A lot of the work that we do relies on the relationships we have with other agencies. If we have good relationships with them then we often are then able to shape the way that our clients and families can access the services that they’ve got*. (Social Worker, Congress)

PHC services were playing a crucial role in joining up the services that are available but which for a range of cultural, social and other reasons were hard for people to access.

#### Advocates for policy change

The PHC services advocated for policy change relating to SDH. The most striking examples uncovered in our study were from the two services with independent boards of management. Congress reported a campaign to increase the unit price of alcohol so that very cheap liquor was unavailable. There had been some success in this and staff reported that there had been a reduction in violent assaults and homicides as a result [54]. Most of their advocacy effort was through a People’s Alcohol Action Coalition with one of Congress’ medical practitioners often acting as a media spokesperson. Congress was also involved in advocacy on achieving suitable and affordable housing for Aboriginal people in Alice Springs where many live in “town camps” with inadequate housing and infrastructure. Congress also advocated to the Federal government for comprehensive PHC and the need for Aboriginal controlled health services, to maintain a focus on health promotion and disease prevention including work on SDH. The overall pattern of advocacy at Congress was described by one of the managers:

*[Advocacy] means that we’re getting the policy environment focused on what we are saying is going to work for Aboriginal people. So it’s about using our capacity and our ability to shape the way in which government decides around health policy or education, any of the health systems, and anything to do with broader social determinants.* (Manager, Congress)

SHine SA also has a clear commitment to advocacy and directly sees this work linked to SDH issues as explained by a manager:

*SHine does a lot of trying to convince other organisations at a national level that we need a national sexual health strategy. We need to do some work around what are the costs of continuing with no intervention around unplanned teenage pregnancy … the cost is actually to those young women who have low levels of health literacy, access to services, who probably have been a child of a single parent who hasn’t worked for 25 years. I mean that’s the social determinant stuff that we need to address. That’s the only way we will change some of the issues*. (Manager, SHine SA)

The organisation had been involved in a high profile campaign to deliver a well-researched sexual health program in schools, which was based on training teachers to teach issues of sexuality and respectful relationships which are important SDH [55]. Opposition from some politicians and religious figures meant the program has often been in the news and staff from the centre had been vocal defenders of the program and its benefits to the health of young people [55]. A further example of advocacy was that the organisation lobbied members of parliament when a bill was proposed that would have raised the age at which young people could access medical treatment without their parents’ consent.

Our study also indicated that PHC workers face barriers in undertaking advocacy. Most significantly, the state-managed services noted the conflict between advocacy and their role as public servants. As a worker at Service B noted:

*If we were advocating for community against something that the government had decided, well then that wouldn’t go down too well.* (PHC Worker, Service B)

Time was noted as a further constraint and especially in terms of the high demand and long waiting lists for services to individuals. Policy advocacy was seen to result from community development work and the state managed services noted that whereas their services had employed several such workers in the past, these positions were being replaced by roles that concerned direct services to individuals. As one worker at Service A said that “limits the advocacy with community that we can do”. The best examples of advocacy we found came from the NGOs in our study and the notion that advocacy was a more acceptable activity for them was borne out by one worker at Service C who noted that when she had previously worked in an NGO advocacy was encouraged and her role had more flexibility and support for community and advocacy work.

### Limits to action

While staff generally showed a considerable awareness of SDH and were able to point to the action they undertook, they also were aware of the limitations on their ability to take action. These limitations were partly about the limited extent of any health sector response to SDH and partly about the constraints placed on action by particular policies or practices within the health sector. The former point was made by a number of respondents and articulated by one of the staff at Service C:

*I don’t know that we do a huge lot on the social determinants of health. I think we’re looking at symptoms, more than causes actually. It’s probably on a higher level than we are, like having someone at the top in health speaking to somebody at the top of transport and speaking to somebody at the top of housing … I think that’s probably a higher kind of level than I’m ever likely to operate at.* (Worker, Service C)

This worker was alluding to the South Australian initiative on Health in All Policies which has been adopted since 2007 [56]. This initiative has been driven by the Public Health Division of the State health department and does not work with the PHC services. Views on the limits placed on action on SDH within the health sector reflect the politics of the health sector at the time of our study. The PHC services that were funded and managed by the State government had undergone significant reorganisation over the five years preceding the research. This reorganisation had seen a shift from separate boards of management (and independent voice) to direct management through regional health structures. This was perceived to have come with a shift in the policy priorities in health so that there was less emphasis on a broad range of social health issues and much more emphasis on chronic disease management and prevention using behavioural models. A regional manager articulated this shift as a concern:

*We would actually be focusing more broadly on social determinants and a more integrated whole of life approach rather than what we are having to do at the moment which is pulling back very much to chronic disease.* (Regional Health Executive)

The impact of the tighter focus in departmental priorities was noted by a number of respondents including this one:

*I think at the moment we’re becoming more clinically focused because of what the department requires, and so our ability to do that kind of social action, really grassroots community development is very limited* (PHC Worker, Service B)

Some staff indicated unhappiness about the shift in priorities. The more selective approaches were reported as giving little room to respond to broader social determinants**.** As one regional manager noted:

*I mean, part of the health reform is to address the demand, hospital demand, basically. And so I think that that driving factor around chronic disease and hospital avoidance has really been a major focus, which means that we’ve not kept our eye on the ball of that broader context…* (Regional Health Executive)

This picture of service priorities for government managed services is strikingly different to that of the 1990s [57] when South Australian PHC services aimed, in addition to providing treatment to individuals, to “also reach out to change their social, political and economic environments'' (p. 162) as a means of improving the health and wellbeing of that community. Congress managers reported that health programs funded by the Commonwealth government were often vertical programs and that they were constantly having to adapt their more comprehensive orientation to these funding requirements:

*As a comprehensive primary health care service we think that what they should be doing is funding comprehensive primary health care rather than vertical programs. … And the way that the government is rolling out the large investment at the moment is pretty much in vertical programs* (Medical Officer, Congress)

The result of this shift in priorities meant that the idea of a comprehensive approach to PHC had moved from one that was broadly accepted by the health department to one where the workers advocating for the approach felt unsupported and unable to do community development work that had once been acceptable. Thus the changing policy environment had created a dilemmatic space which many of the workers found hard to navigate.

A further crucial factor which mitigated against the services undertaking action on SDH was the balance reported by respondents between concentrating on peoples’ immediate need as opposed to taking action on underlying determinants. Immediate clinical needs usually won out. The dilemma and the stress caused by this tension was well summarised by one manager:

*I think we’ve got to try and get a balance within our clinical services around how do we just get ourselves out of the treatment regime? This is stuff that I agonise about continuously. How do we get a balance? How do we take that preventative approach? We’re still seeing people who come through our services that have scabies. We’ve got to be able to have the capacity and the vision I think to go and work at a preventative level because it’s total preventable. 80% of what we see is totally preventable. How do you get the balance between that, knowing that, and moving beyond just clinical treatment?* (Manager, Congress)

Another factor that respondents were very much aware of was that, while there were some limited actions that the services could take to address SDH, they were quite powerless in the face of many of the broader determinants apart from being able to become advocates. Thus one worker noted:

*I think a lot of that happens where we try and be part of networks or projects that try and advocate for that change, but often in the grand scheme of things we have very little control over those things* (PHC Worker, Service B)

In a similar vein one of the SHine SA workers noted

*And it just appals me that we go on concentrating on diseases when we’ve got Big Mac on every corner. It’s such a powerful lobby and nobody tackles that. We’ve spent the last probably 40 years recognising and beginning to fight the cigarettes lobby and it’s just sad that when we talk about community health, we don’t talk about where the food is and who’s getting it and why it’s actually cheaper to go to McDonalds.* (Worker, SHine SA)

The sense of impotence in the face of often overwhelmingly difficult social circumstances was a common theme in the Congress interviews with the following comment typical:

*One of the things that makes my heart sink is to see some of these clients who are young, intelligent, have so much potential and drive themselves, and so much inner strength, and yet are in such awful social circumstances from pressures within their families, or lack of housing, or lack of educational opportunity, that are absolutely trapped* (Medical Officer, Congress)

## Discussion

Despite recognition of the potential role of PHC services in responding to SDH [1,24,58] there has been no delineation of the role or the dilemmas PHC services have to confront in order to be effective actors. Our study has documented some of these dilemmas and also demonstrates the potential of PHC to play a constructive role. Our study has documented that PHC workers in our six case study services were able to respond to the SDH by developing a practice which means that their service provision to individuals is done in a way that recognises and takes into account the limitations placed on people’s agency by the economic and social conditions in which they live. The PHC services also provide services at a community level which both acknowledges the importance of community connectedness to health and also responds to threats to the community’s health. Advocacy for individuals is a powerful way of assisting individuals to gain access to vital economic and social resources which assist them to cope with illness or maintain their health. Policy advocacy is also important in regard to threats to the overall health of communities. Thus we have found that PHC do play a very constructive role in addressing the SDH and also found evidence that they could do more given a strong policy and management mandate. We found that the tensions between selective and comprehensive approaches to PHC are playing out in the six services in our study. The services they offer and strategies they engage in span biomedical, behaviours and social approaches much as described by Labonte [59]. Most of our respondents showed an appreciation of how social determinants affected health and often, in the face of this information, felt somewhat impotent about their potential to respond to these determinants. The communities served by the services were disadvantaged and had heavy burdens of disease that reflected poverty, high unemployment and lack of education. This burden of disease led to very heavy demands for services to treat illness and in this context taking action on SDH can seem like a luxury. Even those respondents who expressed a strong determination to base their work on a social model of health struggled to articulate how they could do this beyond the fact that their practice reflected an appreciation of the structural constraints their clients faced and some advocacy work. This feeling was reinforced by the fact that so many SDH are complex and deeply rooted in structural class and race inequities and the current political economy of Australia.

When our respondents spoke of the limitations to the work they could do on the SDH it was in terms of a complex world where they were struggling to understand the interactions between different determinants, were conscious of the ways in which unfair economic arrangements made their work difficult because of the disadvantage experienced by their clients, and doing this against a backdrop of organisational change. Our interviews indicated that most PHC workers are reflexive in their response to the dilemmas they face in their work in ways similar to the reflexivity Giddens [42] suggests is required of people in the face of the contradictions of late modernity. It also appears that the more the workers approach their work with an appreciation of the deep-rooted, entrenched nature of SDH the more they used reflexive responses. This was most evident in Congress, the Aboriginal community controlled service which understood their work as playing out against the backdrop of Aboriginal dispossession and the consequent low socio-economic status of Aboriginal people and the institutional racism characteristic of broader Australian society. The complexity of the structures within which social determinants play out mean that without this reflexivity it is easier to revert to individualised medical or behavioural strategies in the absence of a more systematic analysis of the structural causes of health inequities. Hoggett, Mayo and Miller [34] examine the dilemmas of development work in the UK in terms of the need for the workers to traverse “dilemmatic space”. The space occupied by PHC is indeed dilemmatic and our study suggests it is becoming more so as health departments reduce PHC to a selective model which focuses almost entirely on services to individuals and does not pay attention to the SDH and their impact across communities. The services are committed to social justice and most staff appreciate the need for changes to people’s living conditions in order to improve health. Yet their hands are tied in most respects and they see their ability to assist clients to make real changes as limited. They understand the importance of a social view of health but work in a broader health system that reinforces a largely bio-medical view. And even for many who see the broader social view, the discourse on SDH itemizes a number of conditions (mimicking behavioural epidemiology and its lists of specific risk factors) rather than presenting a more unified political analysis of how these conditions may be linked, potentially adding to workers’ sense of not being able to make much of a difference. For those working in Aboriginal health services the dilemmatic space is further complicated by a post-colonial world with its legacies of extreme dispossession, racial discrimination and burdensome bureaucratic requirements [60]. A further issue for PHC services is that their managers are relatively powerless in bureaucratic hierarchies compared to their acute service colleagues, which means that their advocacy for a SDH approach is not made from a position of power and influence. This reinforces the importance of supportive policy and a mandate from fund holders to take action on SDH.

It is evident from our study that a comprehensive approach to PHC requires staff with a range of skills. Those focusing on the provision of services to individuals are necessarily more bio-medical and individually focused. Group work is an important aspect of practice and practitioners need skills in planning and running groups. The broader community-wide roles require community educators, community developers and organisers who are best able to respond to SDH. In these roles skills in community organising, policy development and advocacy and campaigning would be vital. It was these later roles that appear to be under question in the state-managed services. Workers fulfilling these roles all reported feeling that their work was not valued and as they left were being replaced by clinical staff.

A minority of workers (primarily those in community development roles) noted that forms of practice that seek to address social determinants soon come to be seen as ‘political’ because they involve challenging entrenched power. These workers reported feeling less able to speak out about health issues when they challenged vested interests and so highlighted the dilemmas of being employed as a public servant in a role which, if done well, requires workers to be vocal advocates. Congress’s commitment to self-determination has to be negotiated with a state that struggles to come to terms with a long history of colonial dispossession and white hegemony. The subsequent lack of education, housing and employment that characterises Aboriginal people in Alice Springs cannot be addressed by a PHC service alone, even though it is able to contribute to reducing historical disempowerment through its community management and advocacy work. In the same way the state-managed PHC services operate in communities where people have experienced intergenerational unemployment, lack of education and live in poverty, reflecting international and national economic trends. These complexities, rooted in societal structures make PHC actors committed to a social view of health almost frozen in the headlights of these overwhelming forces whose causes are far removed from the reach and influence of the PHC services.

One way of dealing with the dilemmatic space in which the PHC workers find themselves is to build alliances and develop PHC as a social movement. This seemed to be happening most at Congress, a key player in the Aboriginal community controlled health movement which has been a political force since the 1970s [8,32,61]. SHine SA was part of a broader movement for sexual health. By contrast the state-managed services’ staff made frequent reference to the limits on their agency to take political stances as public servants. However, in the 1990s and early 2000s government managed services did appear to engage in advocacy to a far greater extent [39,62]. In this period the services, while still funded and managed by the state government health department, had independent boards of management which gave the PHC services the ability to advocate against government policy and reflect community concerns [63]. In the earlier period (1980s-1990s) there was also an active community health movement in Australia which provided a focal point for national and state lobbying for comprehensive PHC based on a social model of health [46]. The National body – the Australian Community Health Association - was defunded in 1996, and with its demise the social movement that had supported the community health sector ebbed away over the following years.

It is worth noting that since these interviews, there has been considerable change in the South Australian PHC landscape, including the Review of Non-Hospital Based Services [64] which has resulted in the state government endorsing comprehensive cuts to health promotion in state managed PHC services. Indications are these reforms will further reduce state managed PHC services’ scope for action on the social determinants of health.

The study is based on case studies which has allowed for detail consideration of six PHC services and contributes to understanding about the nature of practice in relation to action on the SDH. It is also able to show the impact of organisational environments on the operation of the PHC services. Its limitation is that the number of case study sites was relatively small and so the basis for generalisation is limited. The study did not include fee-for-service general practice which, in Australia, solely provides primary medical care and does not have a broader role in relation to SDH.

## Conclusion

Our study suggests that there are tangible ways in which PHC services are able to take the impact of the SDH into account in terms of the style of service delivery and the openness of their service to the communities they serve. The data from our study indicate that in all study sites aside from the Aboriginal controlled health service the organisational climate was perceived as becoming less amenable to action on the SDH than it had been in the recent past. This study is important in providing a nuanced account of the ways in which PHC services are able to respond to SDH, the constraints they face in doing so and the importance of supportive policies and practice. Responses to the SDH require political and social engagement that is not easy within government funded and managed services and the complexity of developing a practice that does offer a response to SDH requires further policy and practice development. In the absence of a supportive political and policy environment, building alliances with communities and social movements may be one strategy services can adopt, but the broadly unsupportive environment makes it very hard for services to act on the Commission on the Social Determinants of Health’s recommendations. Further research on the value of community control of PHC services is warranted by this study’s findings. In addition, research linking effective work on SDH by PHC services which assesses the types of policy, resource and managerial environments that made them possible would also be helpful to future policy development.

## Competing interests

FB was a Commissioner on the Commission on the Social Determinants of Health. The authors declare that they have no other competing interests.

## Authors’ contributions

FB lead the design of the study, contributed to data collection, and lead the analysis and drafting of the manuscript. DL contributed to the design of the study and made critical comments on drafts of the manuscript. TF contributed to data collection, analysis, and drafting of the manuscript. AL contributed to the design of the study and data collection, and made critical comments on drafts of the manuscript. RL contributed to the design of the study and made critical comments on drafts of the manuscript. GJ contributed to the design of the study and data collection, and made critical comments on drafts of the manuscript. All authors read and approved the final manuscript.

## Pre-publication history

The pre-publication history for this paper can be accessed here:

http://www.biomedcentral.com/1471-2458/13/460/prepub
